# Directly interrogating single quantum dot labelled UvrA_2_ molecules on DNA tightropes using an optically trapped nanoprobe

**DOI:** 10.1038/srep18486

**Published:** 2015-12-22

**Authors:** Michelle Simons, Mark R. Pollard, Craig D. Hughes, Andrew D. Ward, Bennett Van Houten, Mike Towrie, Stan W. Botchway, Anthony W. Parker, Neil M. Kad

**Affiliations:** 1 School of Biosciences, University of Kent, Canterbury, Kent, CT2 7NH, UK; 2 School of Biological Sciences, University of Essex, Essex, CO4 3SQ, UK; 3 Central Laser Facility, Research Complex at Harwell, Science and Technology Facilities Council, Rutherford Appleton Laboratory, Didcot, OX11 0FA, UK; 4 Department of Pharmacology and Chemical Biology, University of Pittsburgh Cancer Institute, Hillman Cancer Research Pavilion, 5117 Centre Ave, Pittsburgh, PA, USA 15213

## Abstract

In this study we describe a new methodology to physically probe individual complexes formed between proteins and DNA. By combining nanoscale, high speed physical force measurement with sensitive fluorescence imaging we investigate the complex formed between the prokaryotic DNA repair protein UvrA_2_ and DNA. This approach uses a triangular, optically-trapped “nanoprobe” with a nanometer scale tip protruding from one vertex. By scanning this tip along a single DNA strand suspended between surface-bound micron-scale beads, quantum-dot tagged UvrA_2_ molecules bound to these ‘”DNA tightropes” can be mechanically interrogated. Encounters with UvrA_2_ led to deflections of the whole nanoprobe structure, which were converted to resistive force. A force histogram from all 144 detected interactions generated a bimodal distribution centered on 2.6 and 8.1 pN, possibly reflecting the asymmetry of UvrA_2_’s binding to DNA. These observations successfully demonstrate the use of a highly controllable purpose-designed and built synthetic nanoprobe combined with fluorescence imaging to study protein-DNA interactions at the single molecule level.

A wide range of biological processes crucial to life depend on protein-DNA interactions. These processes include functional activities such as transcription and replication as well as genome packaging, storage and repair. Bulk biochemical assays have provided a core of understanding for these processes; however in recent years single molecule approaches have offered a new means to understand many mechanistic facets inaccessible at the ensemble level. Such approaches use a range of technologies from direct manipulation to fluorescence imaging^1^ to investigate single protein-DNA complexes. Direct manipulation has been primarily conducted through the use of latex or silica spheres optically trapped with laser tweezers or suction pipettes. Single DNA molecules suspended between such spheres have been studied to reveal the mechanical properties of DNA and also the effects of tension on protein-DNA contacts^2^,^3^,^4^,^5^. Alternatively, magnetic tweezers have provided a means to study multiple single DNA strands simultaneously revealing numerous aspects of protein-DNA interactions^6^,^7^.

In parallel to direct manipulation, a number of fluorescence imaging based approaches have been developed. To enable clear interactions to be distinguished, the normally collapsed DNA is elongated using techniques such as DNA combing^8^, DNA curtains^9^ or DNA tightropes^10^. These techniques have enabled the arrival, departure and biochemistry of individual proteins and protein complexes to be imaged, providing new insights into molecular mechanisms for a wide range of protein-DNA systems^10^,^11^,^12^,^13^,^14^,^15^. Meanwhile fluorescence imaging combined with the use of laser tweezers to elongate DNA has provided further technologies to extend our understanding of protein DNA interactions^16^,^17^,^18^,^19^,^20^,^21^,^22^,^23^,^24^,^25^. However, to permit direct manipulation of proteins bound to the DNA the micron-sized spheres used for conventional optical trapping are unsuitable because of their large, imprecise contact area.

Here we exploit the developments in the field of optically trapped microstructures^26^ to develop a new tool that offers the potential to directly probe protein-DNA interactions. We have lithographically fabricated optically trappable ‘nanoprobes’^27^, which are triangular microstructures with a nanometer scale protrusion (Fig. 1a). In combination with DNA tightropes, where single DNA strands are suspended between micron sized platforms, single proteins interacting with DNA have been studied directly. Micro-structured probes have previously been constructed for the potential examination of biological systems both mechanically^28^ and optically^29^ and even naturally occurring diatoms have been used as probes^30^. This study is a proof of concept for the use of such precisely manufactured synthetic nanoprobes in the measurement of a biological system *in vitro* at the single-molecule level. Using this new assay UvrA_2_, a component of the nucleotide excision DNA repair (NER) pathway^31^, has been characterized bound to DNA. We infused UvrA_2_ tagged with quantum dots (Qdot-UvrA_2_) into a microfluidic cell containing pre-assembled DNA tightropes. Following this, an optically trapped nanoprobe was guided to and then swept along a DNA tightrope. Upon encountering a single DNA-bound Qdot-UvrA_2_ molecule the nanoprobe was deflected, showing that we are able to physically interact with a single molecule outside the laser trapping field on a single DNA tightrope. The peak deflections segregated into two populations; we postulate that these may reflect the shape of the Qdot-protein complex bound to the DNA, thus providing a gross structural characterization of the molecule. This study is a demonstration of a technology that could be refined to tackle a wide range of questions, including understanding the structure and/or interaction energetics of proteins with DNA.

## Material and Methods

### Proteins

Biotinylated UvrA was purified and labeled with streptavidin conjugated Qdots (Invitrogen Q10121MP, peak fluorescence emission at 655 nm) as described previously^10^. In the conditions used for labeling we have shown previously that only a single protein is conjugated to each Qdot^10^,^32^. Labeled protein was then diluted to between 1 and 3 nM in 50 mM Tris-HCl (pH 7.5), 50 mM KCl, 10 mM MgCl_2_, 100 mM DTT and 1 mM ATP immediately prior to use.

### Nanoprobes

**Figure 1:**
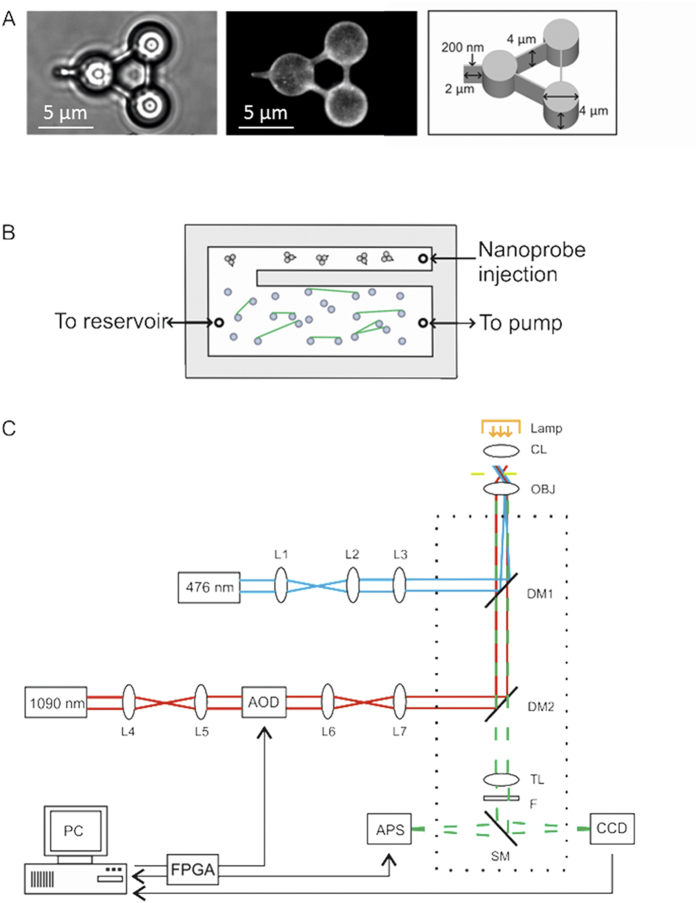
Design overview of nanoprobe, flow cell and optical systems. (**A**) Images of a nanoprobe in solution taken using brightfield (left-hand panel) and fluorescence (middle panel). The right-hand panel is a diagram of a nanoprobe including dimensions of vertex cylinders and tip. (**B**) Schematic diagram of the flow cell showing an upper ‘holding area’ for injection and storage of nanoprobes and a separate lower region where DNA tightropes are constructed (shown in green). (**C**) Diagram of imaging and trapping optics. Optical pathway of 1090 nm trapping laser (red), 476 nm fluorescence excitation laser (blue) and the emission pathway for fluorescence and brightfield (green). L1-3 are beam expansion and focusing lenses. L4-7 beam expander and tube lens, CL condensing lens, OBJ Nikon 1.49NA 60× objective lens, DM1-2 are dichroic mirrors, AOD acousto-optic deflector, TL tube lens, F filter, SM switchable mirror, APS active pixel sensor, EMCCD camera.

Nanoprobes were produced by electron beam lithography (EBL) as described previously^27^. Briefly, a sacrificial layer of chrome was deposited onto a silicon wafer followed by a layer of SU8, which was then selectively exposed to EBL. This produced 4000 repeated nanoprobe structures in one ~0.5 × 0.5 cm square. Each of the structures (Fig. 1) was composed of three 4 μm diameter cylinders connected by struts such that the distance between the cylinder mid-points was 6.8 μm. The nanoprobe height was uniformly 4 μm including the 2 μm (200 nm width) projection from one cylinder that was used to probe the DNA tightropes. This projection offered the advantage of a smaller interaction area and also of ensuring clear separation between the trapping field and the biomolecules in contact with the nanoprobe tip. EBL offers 5 nm accuracy resulting in a nanometer-sharp edge being presented to any proteins attached to the DNA, as confirmed using scanning electron microscopy^27^. Release of the nanoprobes from the silicon was achieved by applying a droplet of chrome etchant (ceric ammonium nitrate) directly onto the wafer. After 30 minutes this was carefully removed without disturbing the probes on the silicon wafer surface. The probes were then gently washed with water multiple times while still on the wafer before finally being removed from the wafer by vigorous pipetting with water. At each stage the post-wash water was pH checked to ensure no carry-over of acid. In addition, the probe square was visually assessed to minimize probe losses during wash stages thus ensuring maximum probe release in the final stage. The probe suspension was then mixed 1:1 with 50 mM Tris-HCl (pH 7.5), 50 mM KCl, 10 mM MgCl_2_, 1 mg/mL bovine serum albumin and 0.01% (v/v) Tween 20 before use.

### DNA tightropes and flow cell design

To permit nanoprobes to be used on demand we constructed a custom flow cell incorporating a main channel for DNA tightrope formation and a side channel for nanoprobe introduction (Fig. 1b). Three fluid entry ports were drilled using a diamond tip drill-bit (Precision Dental), two for the main channel and the third for the nanoprobes. The two channel design was cut from double sided tape (180 μm UK industrial tapes) and sandwiched between a 26 × 76 × 1 mm microscope slide and a 24 × 40 mm #1.5 mPEG_5000_ (N-succinimidyl propionate (Sigma-Aldrich)) blocked cover slip. In the main channel, we created DNA tightropes by flowing bacteriophage lambda DNA (NEB) that had been tandemly ligated (to create long DNA strands) over 5 μm poly-L-lysine coated silica beads using a syringe pump (World precision instruments) as described previously^10^,^33^. The DNA was then visualized by the addition of 150 nM YOYO-1 providing a ratio of ~1:4000 dye:bp, this ensured sufficient tightropes were present. Once the DNA tightropes were constructed and UvrA_2_ was added, no more flow was necessary, therefore all imaging was performed in the absence of flow. The nanoprobes were introduced directly using a 25 μL syringe (Hamilton) to the side channel through a simple adhesive tape re-sealable port, which gave us a reservoir of probes for use throughout the experiment. The flow effects resulting from the introduction of this additional volume did not disrupt the DNA tightropes. Furthermore, more fluid could be passed through the main flowcell chamber without affecting the contents of the side channel. This has the benefit of permitting new protein to be added to the DNA tightropes while keeping nanoprobes protected in the side channel.

### Optical train for microscopy

The combined fluorescence imaging and optical trap setup was built around a Nikon TE2000-S inverted microscope (Fig. 1c). The optical traps were generated by the rapid scanning of a 2.5–5 W 1090 nm wavelength laser beam (SPI Photonics SP 05C 0001) using an acousto-optic deflector (Isle Optics) as described previously^34^. The trapping laser beam was passed through the rear port of the microscope and reflected off a DC950SP dichroic mirror (Chroma) into the microscope objective lens (60× Nikon APO TIRF, NA 1.49). The power in the sample plane was measured as 435 mW with an input power of 5 W, thus each vertex of the nanoprobe experienced one-third of this power. It should be noted that the probing tip remains outside the laser field, which is one of the advantages of the technique we describe. To allow fluorescence imaging, a fiber coupled laser (Becker & Hickl, Output power 3 mW, wavelength 476 nm) was expanded and focused using a standard Nikon total-internal reflection fluorescence accessory with its intrinsic ND filter removed. This beam was coupled into the optical path using a DC500LP filter (Chroma). Detection of emission was achieved through two blocking filters, one for the 476 nm laser (500LP) and one for the trapping laser (900SP). Fluorescence images were collected at the binocular port using an Andor DV888 EMCCD camera in conventional mode. Although the geometry of the nanoprobe ensures that the impingent laser is positioned away from the nanoprobe tip and hence the DNA, some light leakage may possibly result in localized heating effects. However, we found no long term effects measured by stiffness of the nanoprobe after trapping for several hours; we also saw no increased propensity for the tightropes to snap when being probed. These observations suggest that local heating, if present, does not affect the experiment. To make force measurements with sub pico-Newton precision at a rate of 15 kHz, the nanoprobe was imaged at its three trapped points using a custom-built CMOS based active pixel sensor (APS)^34^ connected to the microscope side port. Bright-field illumination for the APS was achieved using a standard halogen lamp. The flow cell and its contents were moved with a single step resolution of ~50 nm using a microscope stage control system (Marzhauser Scan IM 120 × 100). The stage was moved at a speed of 1 μm/s in order to provide smooth motion of the stage; a slower velocity would lead to non-uniform motion due to the momentum of the stage. However, in future studies this would be an important parameter to vary, much as has been performed for protein unfolding experiments^35^.

### Data Analysis

**Figure 2:**
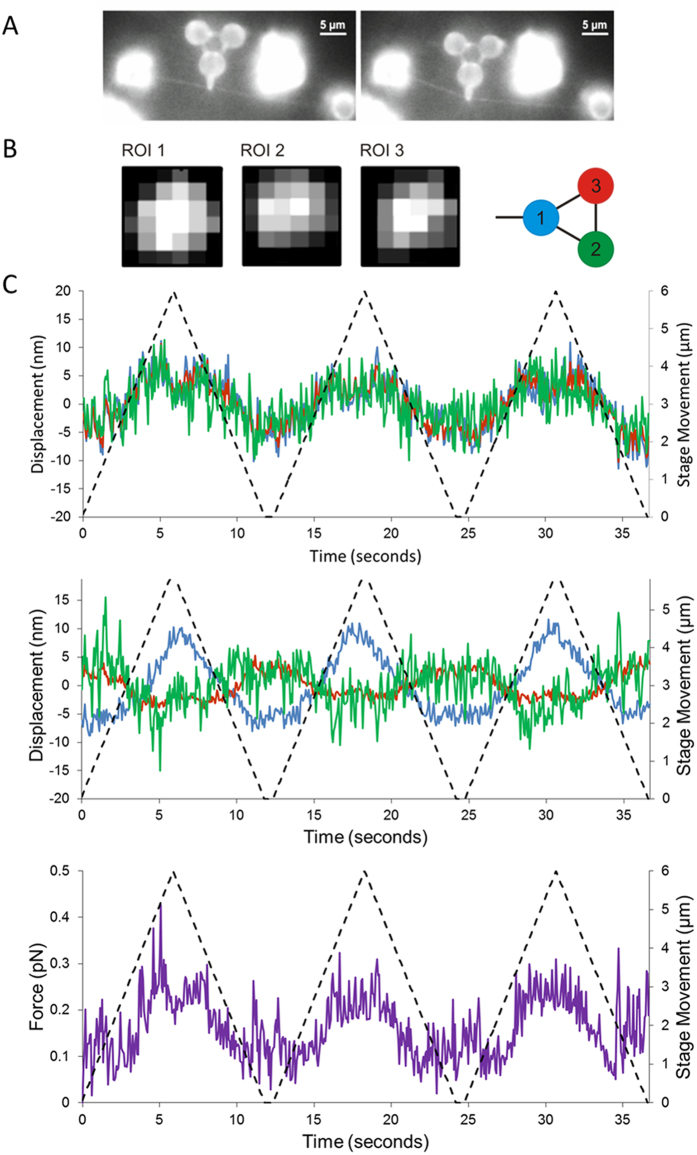
The nanoprobe interacting with DNA not decorated with UvrA_2_. (**A**) OAF images of the nanoprobe interacting with YOYO-1 stained tightropes; in this case the nanoprobe is used to bend a DNA tightrope (see supplementary Movie 2). Further experiments to characterize the bending of DNA are shown in supplementary section 3 and supplementary Figure 2. (**B**) To measure the vertex position of the nanoprobe using the active pixel sensor (APS) three 6 × 6 pixel (417 nm/pixel) regions of interest (ROIs) were centered over the brightfield image of the nanoprobe and data recorded at 15 kHz. (**C**) The centroid position of each point of the probe was calculated in x (top panel) and y (lower panel) to generate a trace of the nanoprobe displacement. The red, blue and green lines represent the three vertices as indicated in (**B**), and the dashed line shows the movement of the stage during each sweep over 6 μm at a rate of 1 μm/sec. In each sweep across the DNA, a very slight response of the nanoprobe is seen, indicating very little non-specific resistance. The lowest panel (purple trace) shows the force experienced at the tip of the nanoprobe.

The data used in this study consisted of images obtained from the APS. Rather than using the whole sensor area, 6 pixel by 6 pixel regions of interest (ROI) on the sensor were selected for improved temporal resolution. Three such regions of interest were positioned, one over each vertex of the nanoprobe (Fig. 2b) and simultaneously sampled at 15 kHz. This rate of data acquisition was just below the maximum response time of the detector (20 kHz^34^) to permit additional control operations, although none were applied in this study. For improved spatial resolution, the position of each cylindrical vertex was then calculated using centroid analysis for each frame in x and y; an example of the centroid calculation in one direction on the image plane (x) is shown in equation 1:


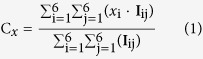


where *x*_i_ is the coordinate of a pixel (i.e. position) on the region of interest and **I**_ij_ is the pixel intensity. To convert the pixel position derived from the centroid analysis into nanometers we used the acousto-optic deflectors to translate the nanoprobe across the ROI in known step sizes.

Prior to each measurement on DNA, the nanoprobe was positioned in a free volume of solution at the same height as the DNA tightropes above the flow cell surface. Any tilting of the nanoprobe, based on bright-field and fluorescence imaging, was corrected for in the optical train of the laser tweezers. The probe’s position variation due to Brownian motion was then measured using the APS and the trap stiffness (*k*_*trap*_) in x and y determined using equipartition theorem for each vertex:


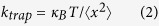


where *κ*_*B*_ is the Boltzmann constant, *T* is the absolute temperature and 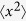 is the variance in that dimension. Since the three nanoprobe vertices are physically connected together in a stiff and fixed formation, the trap stiffnesses for each trapping point were expected to be identical. The values were indeed found to be similar; therefore we used the mean stiffness for each calibration in further calculations. Typical stiffness values were 0.030 pN/nm in x and 0.023 pN/nm in y. This approach provides up to 2 nm spatial resolution^27^. During interactions with DNA the displacement from trap center could be converted into force from the product of the stiffness using Hooke’s law.

## Results

### Scanning DNA with the nanoprobe

The three connected 4 μm diameter cylinders at the vertex of a triangular structure provided handles for manipulating the structure such that contact with the sample could be made via the single rectangular tip (2 μm long by 200 nm wide by 4 μm deep) extending from one cylinder face (Fig. 1a). Optical traps were positioned over each of the three nanoprobe vertices; this permitted fine control over the movement of the nanoprobe (supplementary Movie 1). To measure the position of each vertex of the nanoprobe at high-speed with nanometer accuracy, a CMOS 2D detector array active pixel sensor (APS)^34^ was used. Interactions with immovable objects led to clear tip deflections that could be translated into a resistive force (supplementary Figure 1).

To investigate the interaction of UvrA_2_ with DNA we directly probed the Qdot tagged protein on DNA with the nanoprobe while imaging using oblique angle fluorescence^10^. This technique generates a highly inclined far-field illumination beam by steering the incident laser to a near-critical angle (see Fig. 1c and methods section for a detailed description of the microscope); reducing the background noise from fluorescent particles deeper in solution, but allowing imaging of the tightropes. The nanoprobes were synthesized from the polymer SU8, which has previously been shown to fluoresce at ~530 nm when excited at 485 nm^36^. Therefore we were able to visualize nanoprobes simultaneously with single molecules of YOYO-1 stained DNA and Qdot labeled UvrA_2_ when excited at 476 nm. This coincident imaging was crucial to ensure that the nanoprobe was correctly positioned in focus and just in contact with the DNA prior to scanning for interactions with bound proteins (Fig. 2a). To demonstrate a clear interaction between the nanoprobe and the DNA tightrope we were able to use the nanoprobe to bend DNA as a method for determining the tension (~2.2 pN) on the tightrope (supplementary Figure 2 and Movie 2). Scanning along the DNA for bound proteins was achieved using the automated microscope stage to move the tightrope with the nanoprobe contacting the DNA. Scans were performed multiple times on the same DNA tightrope in either the x or y dimensions (depending on the orientation of the DNA). To measure the resistive force experienced by the nanoprobe tip, the APS sensor system was used. The APS sensor required high levels of visible light for image acquisition at 15 kHz. This prevented simultaneous fluorescence imaging due to filter bleed-through in our setup. Therefore, scans across the DNA were firstly imaged by fluorescence and then repeated using bright-field for APS data collection. The deflection of the nanoprobe was calculated using centroid positioning of each vertex in x and y as it was scanned along the DNA axial contour. In the absence of UvrA_2_ the observed nanoprobe deflections followed the motion of the stage with a magnitude that remained below 20 nm and 0.5 pN (Fig. 2b,c). The symmetry of the peaks for both forward and reverse sweeps show that the mechanical properties of the system are unaffected by repeated scans.

### Measurements of Qdot-UvrA_2_ complexes on DNA

**Figure 3:**
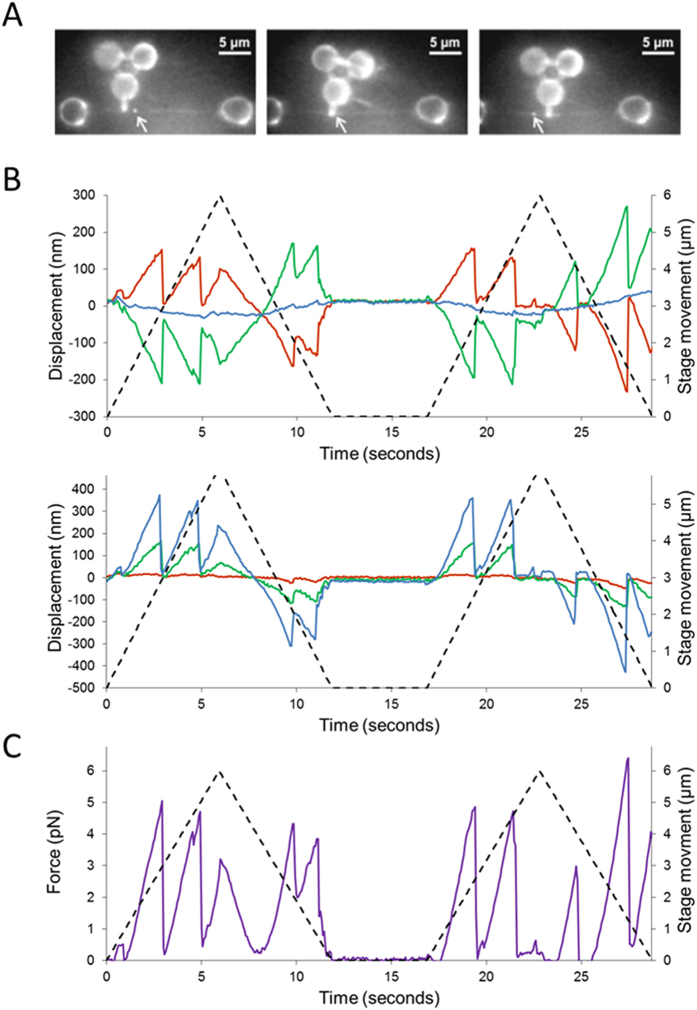
Nanoprobe detection of UvrA_2_ on DNA. (**A**) OAF images of the nanoprobe interacting with a YOYO-1 stained DNA tightrope. The bright spot on the DNA, indicated with an arrow, is a Qdot-labeled UvrA_2_. (**B**) Measurements were made using the APS to generate a displacement trace of the nanoprobe in x (upper panel) and y (lower panel). The red, blue and green lines represent the three vertices as indicated in (Fig. 2B), and the dashed line shows the movement of the stage during each sweep over 6 μm at a rate of 1 μm/sec. (**C**) The displacements were used to calculate the force acting on the bound UvrA_2_ (see supplementary Figure 3). As the nanoprobe sweeps across the protein the resistance increases up to a yield force as the nanoprobe slips past the protein. As the probe performs a return sweep, interactions can be seen in the same location as the forward sweep. This indicates the protein has not detached or moved following the first encounter with the nanoprobe. Further confirmation of this is provided in supplementary Figure 4.

**Figure 4:**
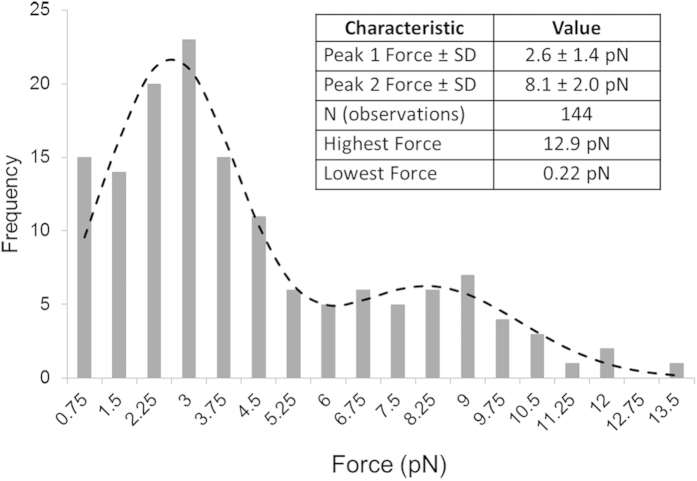
Two distinct peaks for the interaction between UvrA_2_ and DNA predict the molecular mass distribution of UvrA_2_ on DNA. The yield forces from Fig. 3C were plotted as a histogram (n = 144). These data were least squares fitted to the linear sum of two Gaussian distribution functions. The two populations have peak heights of 21.59 and 6.24, peak values of 2.60 pN and 8.13 pN and SD of 1.45 pN and 2.01 pN respectively. The angle of the DNA relative to the nanoprobe had no effect on this distribution (supplementary Figure 6). Table inset: Summary of force nanoprobe Qdot-UvrA_2_ DNA interactions.

When the DNA was scanned with bound Qdot-UvrA_2_, much larger repeatable nanoprobe deflections were observed by fluorescence compared with DNA alone (Fig. 3a and supplementary Movies 3 and 4). High spatiotemporal resolution detection of these nanoprobe interactions was then made using the APS. These deflections were over an order of magnitude greater than in the absence of Qdot-UvrA_2_ (Fig. 3b). Fluorescence imaging showed the presence of a Qdot at the location of the deflection, indicating these data result from direct interactions with Qdot-UvrA_2_. The force acting on the nanoprobe was calculated as the product of the mean scalar displacement from the resting centers of each vertex and the mean trap stiffness, as confirmed previously for this nanoprobe structure^27^. For the measurement of the peak force experienced by the probe tip only, we simplified our system to a single Hookean spring that was extended and then restored to its resting position. The force experienced at the tip is lower than that at the vertices of the nanoprobe therefore a further calculation was performed (see supplementary section 4 for details) to account for this reduction. Each measurement consisted of three to four sweeps (forward and reverse) across the same DNA molecule (Fig. 3b). The resulting force peaks (Fig. 3c; see also supplementary Figure 3) correspond to the resistance offered by the bound protein to the movement of the nanoprobe. Most probe displacements occurred in the same locations on each DNA sweep indicating the protein does not release, nor is not pushed along the DNA by the nanoprobe tip. Therefore the measurements determine the maximum force required to deflect the nanoprobe sufficiently to slip past the bound protein. In total, we measured 144 interactions between the nanoprobe and 36 Qdot-UvrA_2_:DNA complexes. Plotting the peak interaction forces as a histogram yielded two distinct populations (minimal model confirmed by Bayesian information criterion fit to a Gaussian mixtures model – not shown) that were well fit by the sum of two independent Gaussians with means of 2.6 pN and 8.1 pN (Fig. 4).

## Discussion

In this study, we have examined the interaction between UvrA_2_ and DNA, a key step of nucleotide excision repair, using an optically controlled force probe with a protruding nanometer scale tip. The force probe was moved across dsDNA suspended above the surface as tightropes^10^,^15^, and with no protein present there were no deflections. When studied with Qdot tagged UvrA dimers (Qdot-UvrA_2_) bound to the DNA tightropes, the lateral motion of the nanoprobe was impeded. The use of Qdots in these experiments was crucial for locating the bound proteins and providing a larger structure to deflect the nanoprobe. Measurements of the resulting deflections were made using a custom-built high-speed detector providing high spatial precision at 15 kHz, and confirmed using fluorescence detection.

The deflections of the nanoprobe upon encountering DNA bound Qdot-UvrA_2_ complexes resulted in force being applied to the protein. This force increases as the nanoprobe is displaced relative to the protein. Despite forces of up to ~13 pN (Fig. 4) the Qdot-UvrA_2_ was neither moved nor detached. Above these peak forces the nanoprobe rapidly returned to its non-deflected starting position due to either the nanoprobe distorting the DNA and pushing the Qdot-UvrA_2_ around the helix or slipping over the complex. We found positional correlation between the forward and backwards deflections (supplementary Figure 4) suggesting the protein does not slip around the helix, since the protein was still present on the return trip. Therefore our observations are most consistent with the nanoprobe slipping over the top of the protein. The C-terminal conjugation of the Qdots will lead to an accentuation of the asymmetric distribution of molecular mass around the DNA. The asymmetric distribution of molecular mass on the DNA tightrope has been suggested by co-crystal structural data^37^. Since UvrA_2_’s binding to DNA is random, the nanoprobe will sample all axial geometries of DNA-bound UvrA_2_. Therefore, as the probe attempts to pass the Qdot bound region of UvrA_2_ it will experience a greater resistance than when passing the opposite face of the DNA, offering a potential explanation for the two force peaks seen in Fig. 4. However, it may also be possible that the two peaks of the force distribution in Fig. 4 reflect other scenarios such as two configurationally discrete states. UvrA_2_ has been postulated to adopt two distinct structures one of which binds non-specifically to undamaged DNA and the other to damaged DNA^38^. However, given the relatively small structural change relative to that of the Qdot-UvrA-DNA complex it is unlikely that we are resolving these structures. Another scenario is that we are measuring the labeling of UvrA_2_, it is possible that the low force species consists of UvrA labeled with a single Qdot, and the high force population is dual labeled. Due to the brightness and proximity of the nanoprobe we were only able to study the relationship between the number of bound Qdots and peak force amplitude for a limited sub-population of molecules. For these molecules no correlation was found between the number of Qdots associated with UvrA_2_ and the peak force (supplementary Figure 7), suggesting the origin of the two peaks is less likely to be due to the number of Qdots bound to UvrA_2_. Given these arguments we favor the explanation that the nanoprobe is providing a view of the gross structural anatomy of the Qdot-UvrA_2_-DNA complex.

The use of micro-structured probes to study objects has been pursued by a number of groups. Such probes have varied in design and application, including similar triangular structures to measure forces of interaction with colloidal beads^39^, diatoms to measure the topology of cell surfaces^30^,^40^ and more elaborate structures used to measure surface features^28^. The limited application of these devices has been in part due to the difficulty in their manufacture at quantities useful for the study of biological processes^30^ as well as possible direct interference of the trapping laser on the probed object. This study provides details of wafer release coupled with methods to introduce nanoprobes into a flowcell such that 1 in every 2 nanoprobes could be used. Therefore, for a single experiment we were able to obtain data with very low (sub 4000) quantities of nanoprobes. This compares to a typical dual bead laser tweezers experiment^41^ where >1 × 10^6^ beads per flow cell are required in order to capture two useable beads. Alternative devices used to study protein-DNA interactions directly include the use of fixed extruded glass tips. This approach used a larger surface area probe than that used here: a 1–2 μm surface area scanning probe coated with anti-digoxigenin was used to interact with digoxigenin covalently linked to certain DNA bases presented to the tip using optically trapped DNA^42^, recently a similar approach studied proteins interacting directly with DNA extended using magnetic beads^43^. Other approaches include DNA as a probe^23^. This approach used optical tweezers to coil one strand of DNA around the another in a loop to directly detect the restriction endonuclease EcoRI bound to DNA^23^. Movement of the loop along the second DNA strand was obstructed by bound proteins, resulting in displacement of the beads holding the trapped DNA. The bead displacement was used to calculate the force resisting motion on the DNA after subtraction of the DNA compliance. A similar approach has been used to locate EcoRI bound molecules to a magnetically trapped DNA strand^44^. In the study presented here the use of a nanoprobe offers a stiff link between the detector (the probe vertices) and the probe tip, therefore no compliance correction of the probe stiffness is required. With the fine spatial and temporal resolution offered by this system we anticipate its more general application to the study of the energetics of proteins attached to DNA.

This study has delivered a technology combination with extensive potential for studying protein-DNA interactions. However, despite the advances described in this study to make this technology combination possible these experiments remain challenging, future studies will be aimed at simplifying the entire process, bringing down both the levels of expenditure and effort. We envisage that the use of this technique could be further refined by protein conjugation directly to the nanoprobe tip, with such a system it would not be necessary to introduce proteins exogenously to the sample. This has the advantage of permitting the complete energetics of protein-DNA or protein-protein interactions to be assessed. In addition to studying forces, release rates under loaded conditions could also be determined. Finally, the combined approaches laid out in this study could also be used for the study of nanomaterials of biological and non-biological origin.

## Additional Information

**How to cite this article**: Simons, M. *et al.* Directly interrogating single quantum dot labelled UvrA_2_ molecules on DNA tightropes using an optically trapped nanoprobe. *Sci. Rep.* **5**, 18486; doi: 10.1038/srep18486 (2015).

## Supplementary Material

Supplementary Information

Supplementary Movie 1

Supplementary Movie 2

Supplementary Movie 3

Supplementary Movie 4
