# Supplementary material for: Directly interrogating single quantum dot labelled UvrA2 molecules on DNA tightropes using an optically trapped nanoprobe
Source: Sci Rep. 2015 Dec 22;5:18486. doi: 10.1038/srep18486 (PMC4686980; doi:10.1038/srep18486)
Supplement: Supplementary Information [file srep18486-s1.pdf]

Directly interrogating single quantum dot labelled UvrA$_2$ molecules on DNA tightropes using an optically trapped nanoprobe

Michelle Simons[2], Mark R. Pollard[3,5], Craig D. Hughes[2,6], Andrew D. Ward[3], Bennett Van Houten[4], Mike Towrie[3], Stan W. Botchway[3], Anthony W. Parker[3] and Neil M. Kad[1,*]

[1]School of Biosciences, University of Kent, Canterbury, CT2 7NH, UK.

[2]School of Biological Sciences, University of Essex, Essex, CO4 3SQ, UK.

[3]Central Laser Facility, Research Complex at Harwell, Science and Technology Facilities Council, Rutherford Appleton Laboratory, Didcot, OX11 0FA, UK.

[4]Hillman Cancer Center, Program of Molecular and Cell Biology, 5117 Centre Avenue, Pittsburgh, PA, USA 15213

[5]Current address: DFM A/S, Matematiktorvet 307, 2860 K. Lyngby, Denmark

[6]Current address: Department of Veterinary Medicine, University of Cambridge, Cambridge CB3 0ES, UK

[*]To whom correspondence should be addressed: Tel: +44 1227 816151; Email: n.kad@kent.ac.uk

SUPPLEMENTARY MOVIES

SM1: Manipulation of a nanoprobe

SM2: Bending a DNA tightrope with the nanoprobe

SM3: Nanoprobe deflection upon interaction with a single UvrA-Qdot

SM4: Nanoprobe deflection upon interaction with a single UvrA-Qdot

SUPPLEMENTARY INFORMATION

1. **Trapping of cylinders**
2. **Calibration of trapped nanoprobe**
3. **Bending DNA using the nanoprobe**
4. **Forces experienced by the protein**
5. **Supplementary Figures**
6. **Supplementary References**

1. Trapping of cylinders

Experimental and theoretical studies have reported on the trapping of cylindrical microstructures [1,2], where the orientation of the trapped cylinder can be inferred from its dimensions in relation to the focal volume of the trapping laser beam. In our case, we use cylinders of equal length and diameter (4 μm) with a 1090 nm wavelength laser beam focused by an objective lens with numerical aperture of 1.49. The dimensions of the diffraction-limited focal volume were calculated as follows [3]:

In direction of beam propagation, $Z_{min} = \frac{2\,\lambda\,n}{NA^2} = 1.31$ μm

In the transverse direction, the Abbe approximation for a diffraction limited spot gives:

$$r = \frac{\lambda}{2NA} = 0.366\ \mu\text{m}$$

Where NA is the numerical objective, n is the refractive index of water, λ is the trapping laser wavelength.

Previous investigations by the authors on the effects of trapping cylinders of different aspect ratios [4] found that the cylinder of 4 μm size and 1:1 aspect ratio will trap in a stable fashion parallel to the axis of beam propagation. In the experiments reported here we observed stable trapping in both brightfield and oblique angle fluorescence imaging. The nanoprobe was seen to trap in agreement with our previous observations for trapping cylinders. Furthermore, the nanoprobe is trapped at three points that define a geometric plane in which the probe tip is held and is restricted from diffusive movement. Much closer inspection of the movement of the nanoprobe was possible using the active pixel sensor. The positional variance was observed as 137 nm^2 in x and 168 nm^2 in y at standard laser power used in the experiments. Together these data suggest the nanoprobe is stably trapped.

2. Calibration of the optically trapped nanoprobe

Trap stiffness was calibrated when the nanoprobe was held at the same height above the sample cell surface used for scanning DNA and in the vicinity of each suitable DNA tightrope. This was achieved using the equipartition method and cross-checked by comparing the corner frequency of its power spectrum [5]. All power spectra showed that the optically trapped nanoprobe behaved as an overdamped oscillator. For the typical laser power used during these experiments, the escape force for the nanoprobe was measured to be 30 pN, substantially larger than the forces measured during the experiments. Together with previous data [4] that demonstrated the optically trapped nanoprobe exhibits a linear force response and obeys Hooke’s law, we conclude that all measurements were performed well within the tolerance of the system.

3. Bending DNA using the nanoprobe

Using the nanoprobe to bend DNA tightropes led to repeatable deflections (supplementary Figure 1A and supplementary movie 2). Very little deflection perpendicular to the direction of the movement of the probe was observed, suggesting strong homogenous trapping. The nanoprobe was used to

orthogonally push a DNA tightrope of length ~27 µm (supplementary Figure 1A; measured distance between the surface beads) resulting in a ~2.7 ± 0.25 µm deflection; equivalent to extending the tightrope length by ~1.03 µm. In the process the nanoprobe was displaced 211.6 (± 7.7) nm (Supplementary Figure 1B), which corresponds to a measured force of 3.38 (±0.14) pN based on the average trap stiffness (0.027 ± 0.002 pN/nm in x and 0.019 ± 0.007 pN/nm in y). This value of the force required to extend the DNA chain by 1.03 µm was then used to calculate a pretension on the DNA of ~2.2 pN using the worm-like chain extension model for DNA [6]. This pretension is very similar to that modeled previously using a 'ball on a chain' approximation [7], and is consistent with a relative DNA extension of ~90% full contour length. These observations also highlight that the nanoprobe system has the potential to study the mechanics of DNA or other biological or non-biological structures.

4. Forces experienced by the protein

Since the nanoprobe rotates as it interacts with the protein the force applied to the protein contains components both normal to and parallel to the DNA. Furthermore the protein lays at the end of a tip protruding from a triangular structure therefore the protein has a mechanical advantage. This reduces the force experienced by the protein relative to the trap restoring force. To calculate this force we simplify the force diagram to include only a single spring at the top vertex (see supplementary Figure 5a). The centre of rotation is at the centre of mass of this triangle (2/3 height) and therefore as the nanoprobe rotates the top spring is pulled out generating a restoring force F. We can use conservation of energy to calculate the reduction in force at the protein.

$W = F.d$, where F is the force applied by the trap

We want to work out the force applied at the tip, F_t

$y = l.sin60$

where l is the triangle edge length.

For energy conservation the ratio of d to x will define the force at the end of the tip (F_t), thus:

$$^{d}/_{x} = {}^{F}/_{F_t} \ therefore\ F_t = {}^{F.x}/_{d}$$

$$^{x}/_{d} = {}^{y}/_{(y+t)} \ therefore\ F_t = {}^{F.y}/_{(y+t)} = F.({}^{l.sin60}/_{(l.sin60+t)}$$

If $t = 2$ µm, $y = 6.8\sin 60 = 5.9$ µm

$$F_t = {}^{5.9}/_{7.9} \cdot F \ therefore\ F_t = 75\%F$$

In addition, the rotation of the nanoprobe results in force being applied non-parallel to the DNA. This not only produces a normal force but also further reduces the effective parallel force applied to the protein. As shown in supplementary Figure 5b the maximum reduction in parallel force is very small at

<4%. A large magnitude normal force may affect the protein-DNA interaction or the protein's structure. The forces required to unfold proteins typically lie in the several tens to hundreds of piconewtons [8] therefore the maximum normal force applied to UvrA in this study (3.3 pN) is unlikely to affect its structure. Further confirmation of this hypothesis comes from investigating the angle at which the force was applied to the protein on the DNA tightropes by measuring the angle between the nanoprobe and the DNA, which was not always orthogonal. Supplementary Figure 6 shows no angular dependence on the force population suggesting that this is not a factor.

5. Supplementary Figures

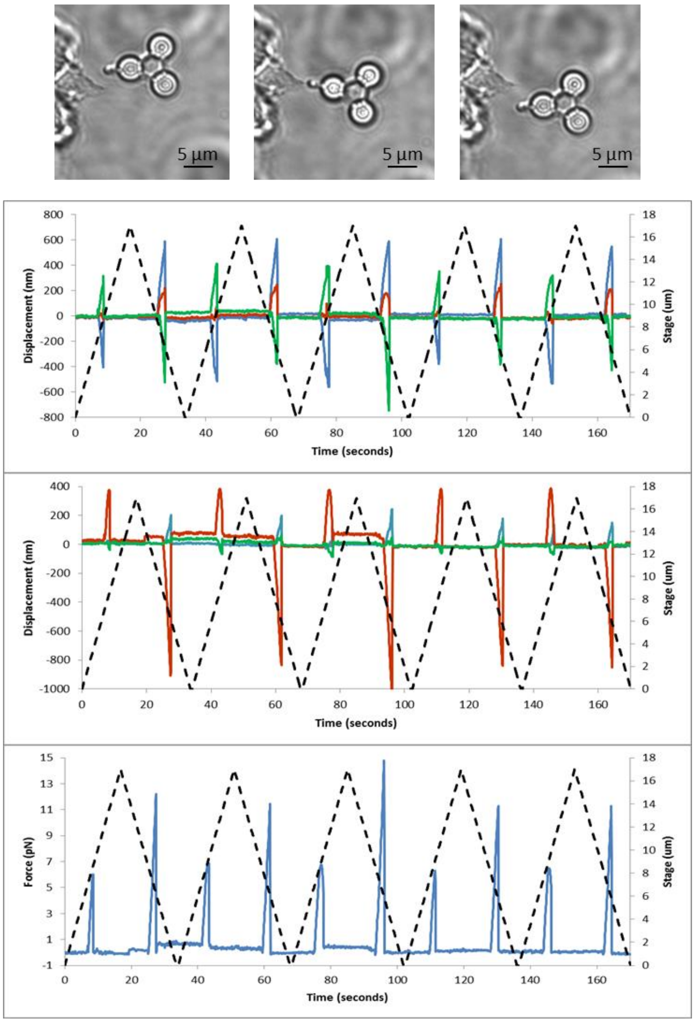

Supplementary Figure 1 – The nanoprobe interacting with a fixed object.

A) Brightfield images of the nanoprobe interacting with a fixed object (a solid glue protrusion at the edge of the flow cell chamber). The nanoprobe is seen to be displaced as the tip contacts the glue. B) Measurements were made using the APS to generate a trace of the displacement of the nanoprobe in x (upper panel) and y (lower panel). The red, blue and green lines represent the three vertices, and the grey line shows the movement of the stage during each sweep over 17 µm at a rate of 1 µm/sec. C) The displacements were used to calculate the force as the nanoprobe sweeps across the protrusion. The resistance increases up to a yield force as the nanoprobe slips past the protrusion, but the protrusion’s shape is not symmetrical and so deflects the nanoprobe by alternating amounts as it scans back and forth, producing the pattern of 6 pN and 12 pN force peaks in Supplementary Figure 1C.

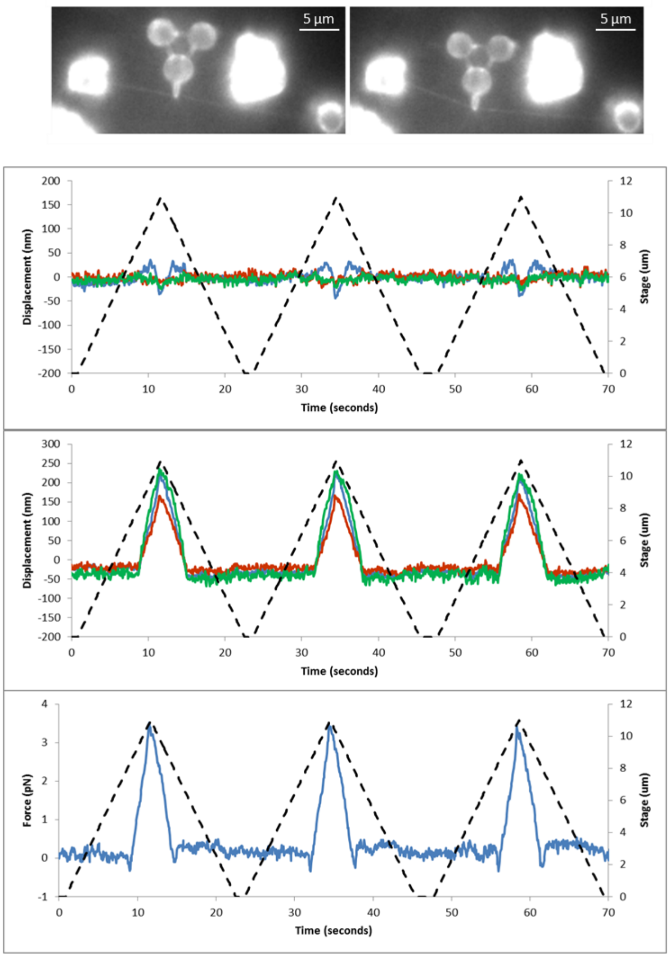

Supplementary Figure 2 – The nanoprobe ‘pushing’ DNA tightropes.

A) OAF images of the nanoprobe interacting with YOYO-1 stained tightropes, in this case the nanoprobe causes a substantial bend in the DNA (this panel is reproduced from Figure 2A). Also, see supplementary movie 2. B) Measurements were made using the APS to generate a trace of the displacement of the nanoprobe in x (upper panel) and y (lower panel). The red, blue and green lines represent the three vertices, and the grey line shows the movement of the stage during each push over 12 µm at a rate of 1 µm/sec. C) The displacements were used to calculate the force acting on the DNA tightrope.

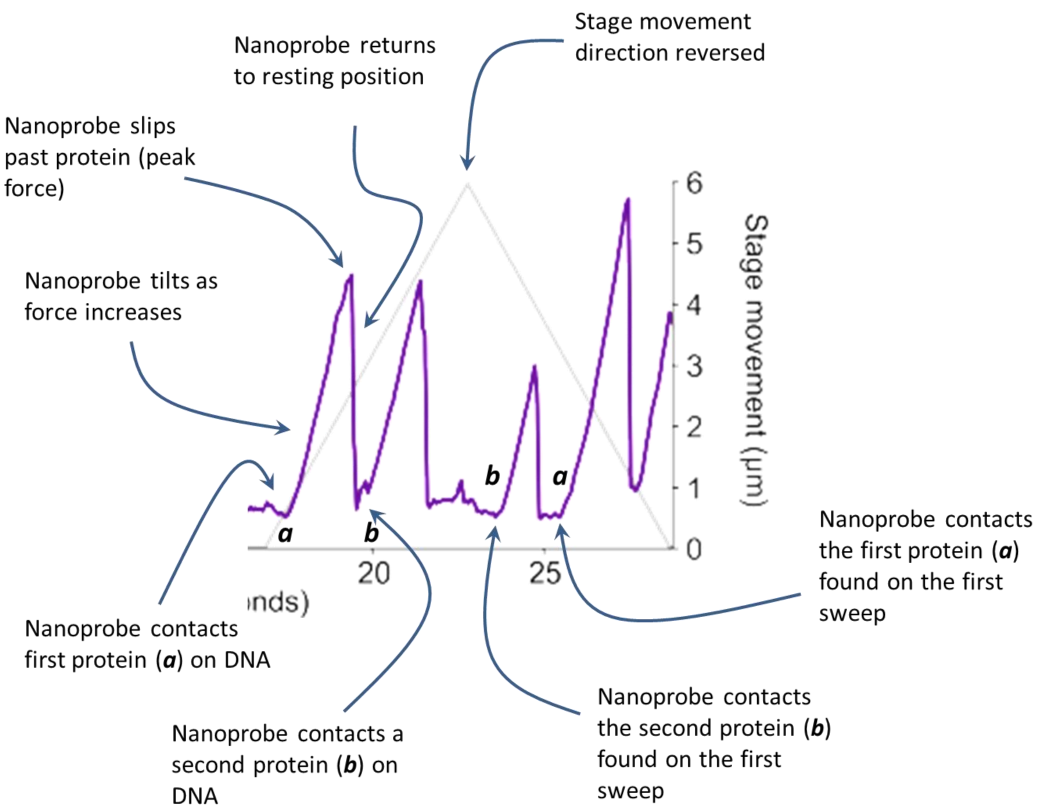

Supplementary Figure 3 – Interpretation of force traces.

This figure is a reproduction of a section of Figure 3c from the main manuscript, however here we have annotated the various features of the force plot to aid in understanding the deflection of the nanoprobe upon interaction with $UvrA_2$ bound to the DNA.

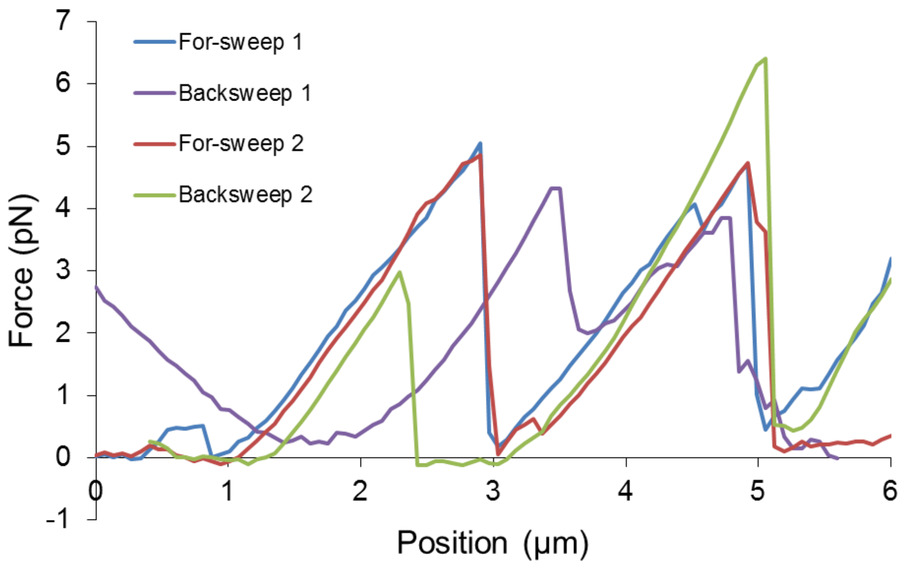

Supplementary Figure 4 – Overlay of nanoprobe sweeps with a displacement abscissa.

Re-plotting the data of Figure 3 using the stage position as an abscissa allows for the position of the forward and backwards sweeps to be correlated. The blue and red forward sweep traces show the high degree of reproducibility for the nanoprobe. In addition, the backwards sweeps also show a high degree of correlation with the forward sweeps. The outlying backwards sweep (purple line) shows a lack of end-point correlation for the first protein interaction but the second interaction overlays again well. This is likely due to the stage direction switch prior to the nanoprobe slipping past the protein. These data confirm the fluorescence observations that the nanoprobe does not appear to reposition the protein on the DNA.

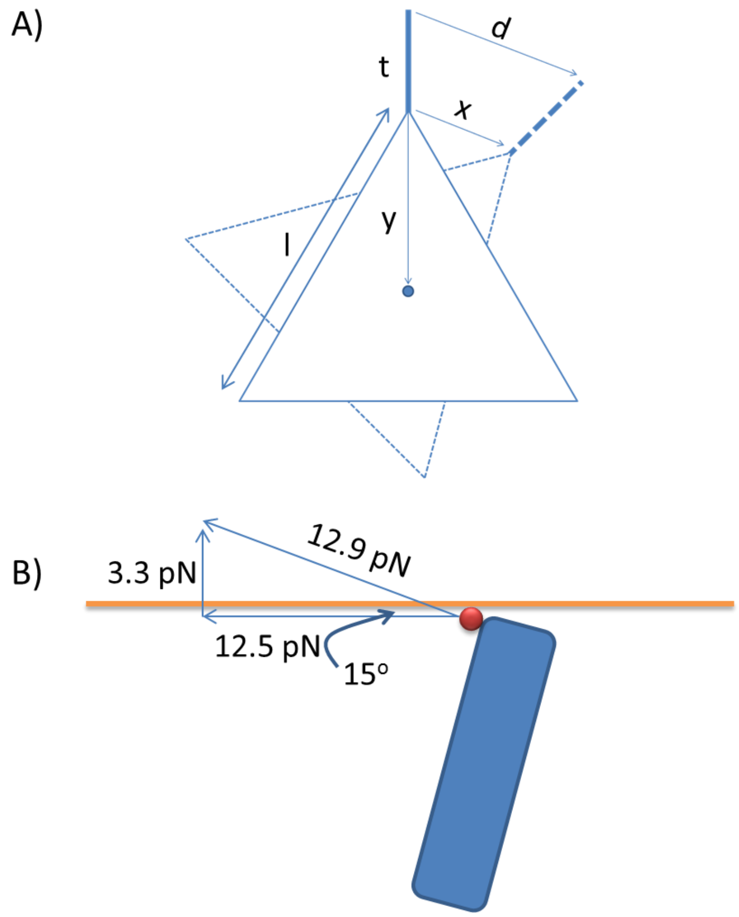

Supplementary Figure 5 – Force application to the protein through rotation of the nanoprobe.

A) The nanoprobe is depicted as a triangle. The center of mass for a triangle held at each vertex corresponds to 2/3 its height. Rotation of the nanoprobe occurs on encountering a protein on the DNA, this extends a spring at each vertex but simplified here to exist only at the top vertex. This spring is extended by x nm but the protein lies at the end of a tip of length, t. Therefore the protein experiences less force than measured because the displacement of the contact at the tip is d nm. B) At full deflection the nanoprobe (blue bar) exerts a force on the protein (red dot), this can be broken down into components parallel to and normal to the DNA strand. It is seen that at the highest force observed in this study (12.9 pN) the parallel component is reduced by <4% due to the angle (15°) of force application.

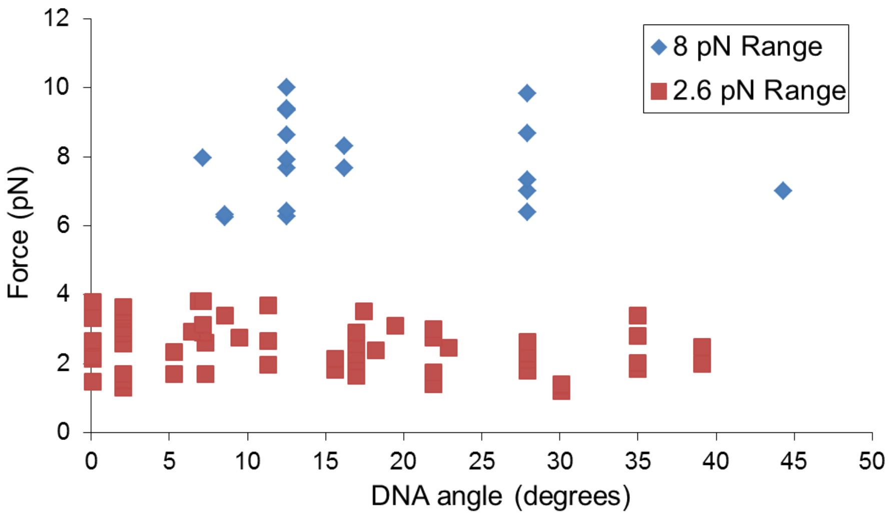

Supplementary Figure 6 – DNA tightrope angle does not affect force measured.

To determine if the two force populations in Figure 4 (main text) were derived from an artifact due to the orientation of the DNA tightropes relative to the stage motion we re-plotted the forces within one standard deviation point of the mean of each population from Figure 4 against the angle of the DNA tightrope during the measurement. The angle of interaction clearly had no effect on the measured resistive force, in either the low or high force regimes.

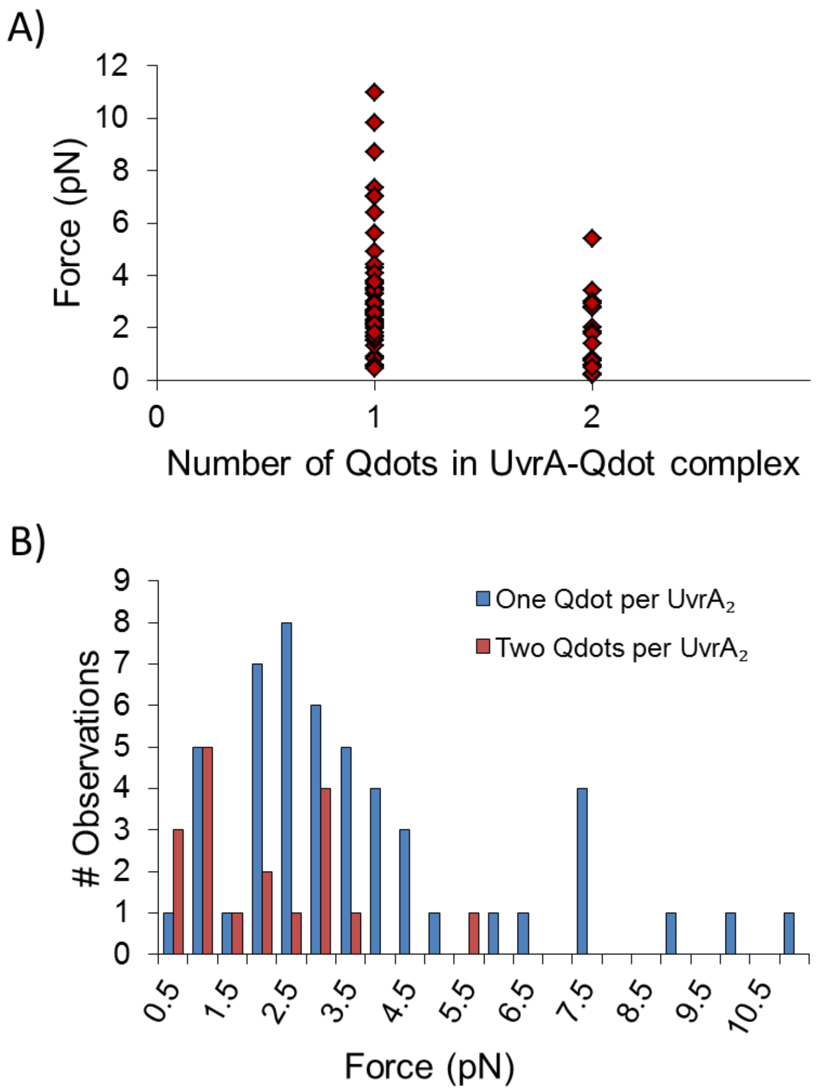

Supplementary Figure 7 – Dual labelled $UvrA_2$ complexes do not elevate force resistance.

To determine if the bimodal force profile in Figure 4 derives from one or two Qdots bound to $UvrA_2$ we examined the fluorescence profiles of the 17 Qdot-$UvrA_2$ complexes that were amenable to fluorescence investigation. Twelve molecules blinked to background fluorescence over the period of examination suggesting the presence of a single Qdot. The remaining 5 complexes fluctuated between two clear fluorescent states suggesting the presence of two Qdots. Because multiple sweeps of the nanoprobe were performed we obtained 68 peak force measurements for these 17 molecules. A) The peak forces (n = 50) for those molecules characterized as single Qdot-$UvrA_2$ species are distributed across the range of forces measured in this study. For those species that were characterized as containing two Qdots, the range of peak forces observed (n = 18) appeared skewed towards lower peak forces. However, given the small number of observations this is not considered significant. Even so, it is apparent that two Qdots bound to $UvrA_2$ do not result in an increased peak force. Caution must also be taken in interpreting the intensity of Qdots since their blinking occurs

across decades of timescales, thus leading to differential brightness when averaged in the image exposure time. B) Re-plot of the data from *(A)* but as a histogram for comparison with Figure 4.

6. Supplementary References

1 Deufel, C., Forth, S., Simmons, C. R., Dejgosha, S. & Wang, M. D. Nanofabricated quartz cylinders for angular trapping: DNA supercoiling torque detection. *Nat Meth* **4**, 223-225, (2007).

2 Gauthier, R. C., Ashman, M. & Grover, C. P. Experimental confirmation of the optical-trapping properties of cylindrical objects. *Appl Opt* **38**, 4861-4869, (1999).

3 Pawley, J. B. *Handbook of biological confocal microscopy*. 2nd edn, (Plenum Press, 1995).

4 Pollard, M. R. *et al.* Optically trapped probes with nanometer-scale tips for femto-Newton force measurement. *New J Phys* **12**, (2010).

5 Florin, E. L., Pralle, A., Stelzer, E. H. K. & Horber, J. K. H. Photonic force microscope calibration by thermal noise analysis. *Appl Phys a-Mater* **66**, S75-S78, (1998).

6 Perkins, T. T., Smith, D. E., Larson, R. G. & Chu, S. Stretching of a single tethered polymer in a uniform flow. *Science* **268**, 83-87, (1995).

7 Kad, N. M., Wang, H., Kennedy, G. G., Warshaw, D. M. & Van Houten, B. Collaborative dynamic DNA scanning by nucleotide excision repair proteins investigated by single-molecule imaging of quantum-dot-labeled proteins. *Mol Cell* **37**, 702-713, (2010).

8 Forman, J. R. & Clarke, J. Mechanical unfolding of proteins: insights into biology, structure and folding. *Curr Opin Struct Biol* **17**, 58-66, (2007).
